# Clinical Practices Following Train-The-Trainer Trauma Course Completion in Uganda: A Parallel-Convergent Mixed-Methods Study

**DOI:** 10.1007/s00268-023-06935-4

**Published:** 2023-03-05

**Authors:** Zeyu Tang, Derick Kayondo, Sarah J. Ullrich, Martha Namugga, Peter Muwanguzi, Gregory Klazura, Doruk Ozgediz, Mari Armstrong-Hough

**Affiliations:** 1 Yale University School of Medicine, 333 Cedar St, New Haven, CT 06510 USA; 2 Faculty of Medicine, Mbarara University of Science and Technology, P.O Box 1410, Mbarara, Uganda; 3 Makerere University College of Health Sciences, P.O Box 7072, Kampala, Uganda; 4 Department of Surgery, Loyola University Stritch School of Medicine, 2160 S 1st Ave., Maywood, IL 60153 USA; 5 Department of Surgery, University of California San Francisco School of Medicine, 533 Parnassus Ave., San Francisco, CA 94143 USA; 6 Department of Social and Behavioral Sciences, Department of Epidemiology, New York University School of Global Public Health, 726, Broadway, New York, NY 10012 USA

## Abstract

**Background:**

Despite the growth of trauma training courses worldwide, evidence for their impact on clinical practice in low- and middle-income countries (LMICs) is sparse. We investigated trauma practices by trained providers in Uganda using clinical observation, surveys, and interviews.

**Methods:**

Ugandan providers participated in the Kampala Advanced Trauma Course (KATC) from 2018 to 2019. Between July and September of 2019, we directly evaluated guideline-concordant behaviors in KATC-exposed facilities using a structured real-time observation tool. We conducted 27 semi-structured interviews with course-trained providers to elucidate experiences of trauma care and factors that impact adoption of guideline-concordant behaviors. We assessed perceptions of trauma resource availability through a validated survey.

**Results:**

Of 23 resuscitations, 83% were managed without course-trained providers. Frontline providers inconsistently performed universally applicable assessments: pulse checks (61%), pulse oximetry (39%), lung auscultation (52%), blood pressure (65%), pupil examination (52%). We did not observe skill transference between trained and untrained providers. In interviews, respondents found KATC personally transformative but not sufficient for facility-wide improvement due to issues with retention, lack of trained peers, and resource shortages. Resource perception surveys similarly demonstrated profound resource shortages and variation across facilities.

**Conclusions:**

Trained providers view short-term trauma training interventions positively, but these courses may lack long-term impact due to barriers to adopting best practices. Trauma courses should include more frontline providers, target skill transference and retention, and increase the proportion of trained providers at each facility to promote communities of practice. Essential supplies and infrastructure in facilities must be consistent for providers to practice what they have learned.

**Supplementary Information:**

The online version contains supplementary material available at 10.1007/s00268-023-06935-4.

## Introduction

Injuries are responsible for more than 5 million deaths annually, and 90% occur in low- and middle-income countries (LMICs) [1, 2]. The disproportionate burden in morbidity is partially attributable to a systemic lack of capacity to manage injured patients; most LMICs lack nationally available advanced trauma training [3, 4].

Context-appropriate trauma training interventions seek to fill this gap. Several successful initiatives have expanded their efforts through train-the-trainer (TTT) programs whereby participants learn to train other providers [4]. The TTT model is attractive because it is inexpensive, enables rapid scale-up, and can increase local ownership of training [5]. However, few LMIC trauma courses demonstrate improvements in clinical outcomes [5–7]. Studies assessing trauma courses typically use surrogate measures such as standardized tests and self-reports, but these instruments may not reflect true practice of skills and are susceptible to response biases [8–11]. We sought to understand whether a TTT program in Uganda influenced patient care using mixed-methods.

## Materials and methods

### Setting

Uganda is an LMIC in sub-Saharan Africa with an injury mortality rate of 90.4/100,000—nearly double that of HICs [11]. In 2007, local and international content experts developed the Kampala Advanced Trauma Course (KATC), a context-appropriate, open-source trauma course, and taught the course to intern physicians at Uganda’s national referral hospital [9]. In 2018, KATC adopted a TTT model and expanded across the country [12].

### Trauma training

Physicians, physicians-in-training, and nurses (KATC-trained providers, KTPs) attended a two-day training at one of three tertiary public health centers [9]. A subset of 8 providers participated in a trainer-specific curriculum on Day 3 and then trained a group of training-naïve intern physicians on Days 4 and 5.

### Study design and data

We followed KTPs from July to August 2019 using a convergent, three-component mixed-methods design: structured real-time observations of trauma resuscitations, in-depth interviews with KTPs, and resource availability surveys.

### Resuscitation observations

We designed an observation tool to measure provider behaviors during resuscitations drawing on the KATC curriculum, World Health Organization (WHO) *Trauma Care Checklist,* and local and international expert consensus (Supplemental Materials S1) [13]. We observed resuscitations in four tertiary care centers where KTPs were employed for 5 consecutive days each (three publicly funded, one privately funded, and one not-for-profit center.) Authors also spent a total of 10 days at district-level facilities but did not encounter traumas in that time. Data were recorded using Qualtrics XM™ (Provo, UT, USA).

### Interviews

ZT and DK conducted in-person, semi-structured interviews with KTPs. All interviews were in English. Interviewers probed trauma practices, perceptions of barriers to care, reflections on the training experience, and experiences incorporating course skills into routine practice (Supplementary Materials S2).

### Surveys

Interview respondents reported access to trauma resources through a semi-quantitative survey adapted from a survey used in other LMICs and the WHO *2004 Guidelines for Essential Trauma* (Table 1)[14, 15].

**Table 1:** **Criteria** for resource access ranking

Score	Meaning	Description
0	Absent	This service or item is never available when needed
1	Inadequate	Less than half of those who need this service or item receive it
2	Partially adequate	Most, but not all, of those who need this service or item receive it when needed
3	Adequate	Virtually all of those who need this service or item receive it when needed

We modified the original resource access tool from Mock et al. [15] to include improvised equipment. The questionnaire instructed respondents to consider access to a specified resource or any improvised equivalents they may use at their facility

### Analysis

We categorized medical actions as equipment-dependent or equipment-independent and recorded the time elapsed between initial patient presentation and a medical action. We plotted time-to-event data as cumulative incidence curves (Prism v9.1.2, GraphPad Software, California USA). We censored actions that were not performed within one hour after the last recorded intervention or evaluation. Observations without timestamps were excluded from time-to-event analysis but included in frequencies and proportions of medical actions performed.

We used an inductive grounded theory approach to code and identify key themes from interviews (Dedoose, CA, USA). We reported equipment survey data as medians, ranges and grouped by facility type (public tertiary, private not-for-profit, district). Finally, we compared observational, interview, and survey data in a convergent analysis to triangulate themes.

### Ethics

The School of Medicine Research and Ethics Committee at the Makerere College of Health Sciences and Human Investigation Committee at Yale University approved the study. Facility directors gave written approval to conduct interviews and observe trauma resuscitations. Each respondent provided verbal informed consent.

## Results

### Real-time observations

We observed 23 resuscitations (Table 2). Patients were mostly male (78%), within the 24–44 age group (70%), and had head injuries (74%). The most common causes were road traffic injuries (48%) and interpersonal violence (35%). A median of 2 providers participated in each resuscitation (range 0–8); more providers participated in each resuscitation at the private facility (5) compared to public facilities (2). In the private facility, KTPs led every resuscitation (3/3). In public facilities, only one resuscitation was led by a KTP (5%). Two unconscious patients at public facilities were never evaluated or treated before leaving or being carried out by laypersons.

### Equipment-Iindependent practices

**Fig. 1:**
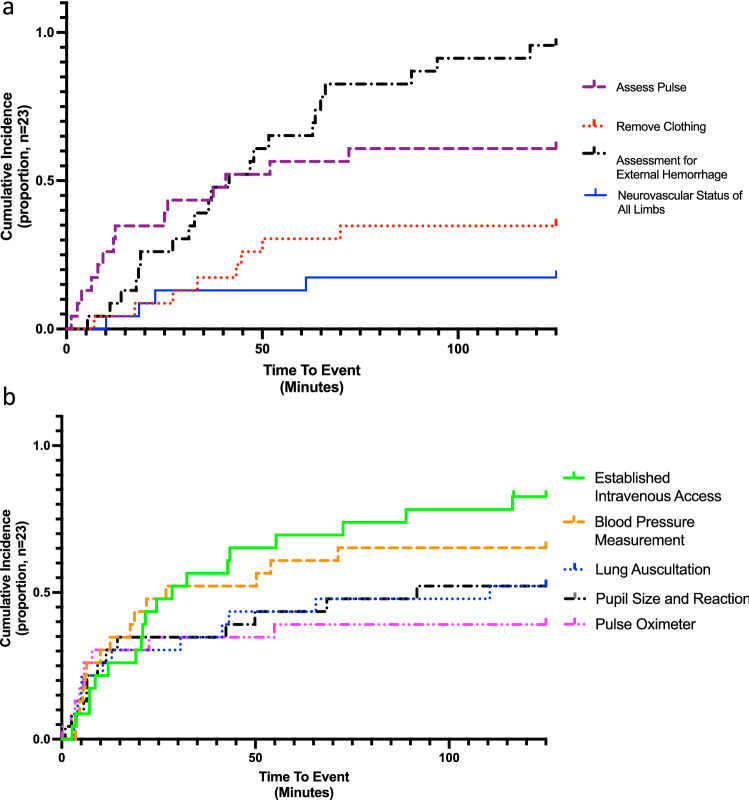
Time-to-event plots of observed resuscitations. **a** Equipment-independent actions, **b** observations of behaviors that required equipment. Out of 19 instances where providers established intravenous (IV) access, 10 were established with one peripheral IV instead of two

Equipment-independent practices were inconsistently applied and varied greatly in time-to-event (Fig. 1a). Pulses were checked in 61% of cases with a median wait of 12 min. Evaluation for external hemorrhage almost always occurred (96%, 39 min). However, most providers limited external injury examinations to the head and exposed distal limbs. Removal of clothing (35%, 38 min) and assessment of neurovascular status (17%, 47 min) were infrequently performed. Providers used at least one component of the AMPLE history (Allergies, Medications, Past illnesses or Pregnancy, Last meal, and Events related to injury) [16] in 83% of patients, but this was almost always limited to events.

**Table 2:** Characteristics of Trauma patients

Patient	Gender	Age	Tertiary hospital	Mechanism of injury	Location of injury	Number of staff involved	Notes
1	Male	15–24	Public	Road traffic injury	Head, arms	0	
2	Male	24–44	Public	Interpersonal violence	Head, arms	2	
3	Male	24–44	Public	Interpersonal violence	Head	2	
4	Male	24–44	Public	Interpersonal violence	Head, arms	0	
5	Male	24–44	Public	Interpersonal violence	Head, legs	0	
6	Male	24–44	Public	Road traffic injury	Head	5	
7	Male	15–24	Public	Interpersonal violence	Head, arms	4	
8	Male	24–44	Public	Road traffic injury	Head, legs	1	33 min to make a cardboard splint
9	Female	15–24	Public	Road traffic injury	Abdomen, Legs	5	5 min to create improvised wound packing material from gauze

### Equipment-dependent practices

We assessed five universally applicable equipment-dependent actions (Fig. 1b). Pulse oximeters were placed in 39% of patients (median 6 min); lung auscultation (52%, 12 min) and pupil examinations (52%, 10 min) performed in half; and intravenous (IV) access established in most (83%, 22 min). Measurement of blood pressure (BP) with a cuff occurred in 65% of cases (13 min). Seventy percentage patients received a BP measurement or pulse check.

Providers in public facilities performed fewer equipment-dependent actions. Pulse oximetry (30%) and airway auscultation (45%) were performed less than half the time. Although IV access was established in most resuscitations (80%), most (63%) received one IV instead of two. Once, a single pediatric IV catheter was used for an adult because larger sizes were unavailable. In contrast, private facility providers performed pulse oximetry, airway auscultation, dual large-bore IV access with adult-sized catheters for all resuscitations observed.

In public facility resuscitations, providers spent > 10 min gathering or improvising for missing resources; four were delayed > 25 min. Private facility providers did not spend additional time gathering equipment.

### Interviews

We invited 29 KTPs to in-depth interviews; all agreed but two were unavailable at the time of visit (Table 3). Most (66%) were male. Most respondents were medical officers (44%) or nurses (33%), with a median 5 years of clinical experience. Additional excerpts for themes are available in Supplemental Materials S3.

**Table 3:** Demographics of interview participants

	Tertiary public hospitals	Not-for-profit private hospitals	District-level hospitals	Total
Number of interviews	14	4	9	27
Female gender	6	0	3	9
Qualification				
Medical officer	5	4	3	12
Nurse	4	0	5	9
Specialist surgeon	3	0	0	3
Senior house officer	2	0	0	2
Medical clinical officer	0	0	1	1
Surgical volume per month				
0–100	4	1	7	12
101–200	2	2	1	5
201–300	4	1	0	5
301–400	0	0	1	1
401–500	1	0	0	1
More than 500	3	0	0	3
Injured patients per week				
0 to 10	0	0	3	3
11–20	1	1	3	5
21–30	1	1	1	3
31–40	1	0	0	1
41–50	1	0	0	1
More than 50	11	2	1	14
Trauma-related procedures per week				
0 to 10	0	0	3	3
11–20	1	3	2	6
21–30	1	0	0	1
31–40	1	0	1	2
41–50	3	0	0	3
More than 50	6	1	0	7

#### Positive impression of training

KTPs reported that the trauma course transformed their practices and self-perceptions. Respondents felt their previous medical education did not sufficiently address the high burden of injuries in real practice. One respondent (nurse, tertiary public) felt the course improved basic skills, “especially in triaging who’s most sick, who needs urgent attention.” A few gave examples of when they believed KATC training helped them save lives. All respondents thought advanced trauma training should be widely available, and most asked for KATC to train their colleagues.

#### Forgetting

All respondents noted unanticipated difficulties applying knowledge and skills in real clinical scenarios. Respondents most frequently cited *forgetting* as a major barrier. Reflecting on the course itself, most described the amount of information and pace as overwhelming. Many wanted more time dedicated toward “hands-on” skill training. All wanted routinely scheduled refresher courses to review complex or infrequently used skills.

One medical clinical officer (district-level) explained that KTPs are less willing to use trauma training in real clinical situations if they are apprehensive of their competency, and disuse may cause them to forget entirely over time. A frequent and widespread concern among respondents was the loss of motor skills (e.g., placing chest tubes), though only a few described problems with facts or mental frameworks; district-level providers were specifically worried about insufficient trauma volumes at their facilities to sustain motor skills.

#### Isolation

Many respondents felt that training one to three providers from each facility did not fundamentally change trauma practices; one respondent described KTPs as “islands of knowledge.” Respondents found teamwork challenging because non-trained providers were unfamiliar with the concepts, roles, and workflow. Yet not enough KTPs were trained at any facility to form teams on their own. Respondents unanimously suggested training more providers from each facility so they could rely on each other and change the practice culture. Respondents’ experiences sharing knowledge with non-trained providers were limited. Most described brief, informal sharing of specific knowledge or skills. One respondent (nurse, district-level) organized a training workshop for her facility and thought others appreciated the training.

Respondents felt they could not permanently change the practices of non-trained providers on their own. One medical officer (district-level) was uncertain if his colleagues used the training after he went home, even if they showed signs of adoption during his shift. In teaching hospitals, a medical officer observed gaps in coverage related to intern physician training schedules: “when you pass on the skill to those very doctors… after four or three months, they’re gone.”

#### Resources

All respondents highlighted a widespread lack of trauma care resources—such as diagnostic, interventional and teaching aids—as significant barriers to changing practices at their facility. Respondents felt that equipment shortages made application of KATC skills more difficult. One senior house officer (tertiary public facility) felt frustrated by systemic problems: “[trauma training] can help at the moment when you are the doctor attending to this patient that has come in, but as in improving trauma care generally, it almost doesn’t help.”

### Survey

All 29 participants completed resource availability surveys (Supplemental Materials S4). Private facility providers reported greatest access to trauma resources, followed by district-level providers (Table 4). Public tertiary providers reported the lowest access.

**Table 4:** Select findings from resource availability survey

Comment	
Patient monitoring and diagnostics	Providers at private hospitals had access to pulse oximeters, in contrast to all other facilities. All respondents reported a lack of access to capnography, esophageal detectors, central venous pressure monitoring. Providers at private hospitals had access to diagnostic capabilities such as arterial blood gases, lactate, measurements, but public providers did not
Interventional equipment	Providers from one tertiary public hospital and both private hospitals reported the highest availability of pediatric endotracheal tubes, laryngoscope handle and Macintosh blades, and chest tubes. Although all three facilities reported access to underwater seal bottles, tertiary public providers almost always improvised the bottles from plastic crystalloid containers while private hospitals did not improvise. Magill forceps, pediatric-size oropharyngeal airways, stiff suction tips, mechanical ventilation, central venous lines, electric cardiac monitoring, fluid warmers, pressors, cervical collars, spine backboard, intraosseous needles were mostly or always available in providers in private facilities, but not to public providers Cricothyroidotomy sets, capabilities for right-heart catheterization, and pediatric Magill forceps were absent for nearly all providers
General and safety equipment	Providers at private hospitals had access to practice guidelines for emergency care, basic trauma packs, and eye protection. Protective equipment were always available (3) in private hospitals. Protective equipment in tertiary public facilities were most commonly improvised or reused. For example, nurses reused disposable gowns and made sharps disposal containers from cardboard boxes Providers from one hospital (private) had weighing scales

### Integrative analysis

We observed that tertiary public facilities lacked basic trauma resources. Non-consumable equipment such as bag-valve masks, BP cuffs, pulse oximeters, and stethoscopes were rarely available. In interviews, public facility KTPs explained that equipment were often lost, locked away, or stolen. In comparison, observations, interviews, and surveys of private facility KTPs demonstrated consistent access to resources; dedicated equipment was present within the resuscitation area.

Public tertiary facilities had few providers on duty and high volumes of injured patients (Table 3). Frontline providers carried out initial assessments and resuscitations alone or in pairs. Most were nurses or rotating physicians-in-training; none were KTPs. We observed only one instance of KTP-led resuscitation among public facilities. In contrast, the private facility permanently staffed KTPs as frontline providers on dedicated teams of 4–8 providers. There, KTPs coordinated the team to complete tasks simultaneously.

**Fig. 2:**
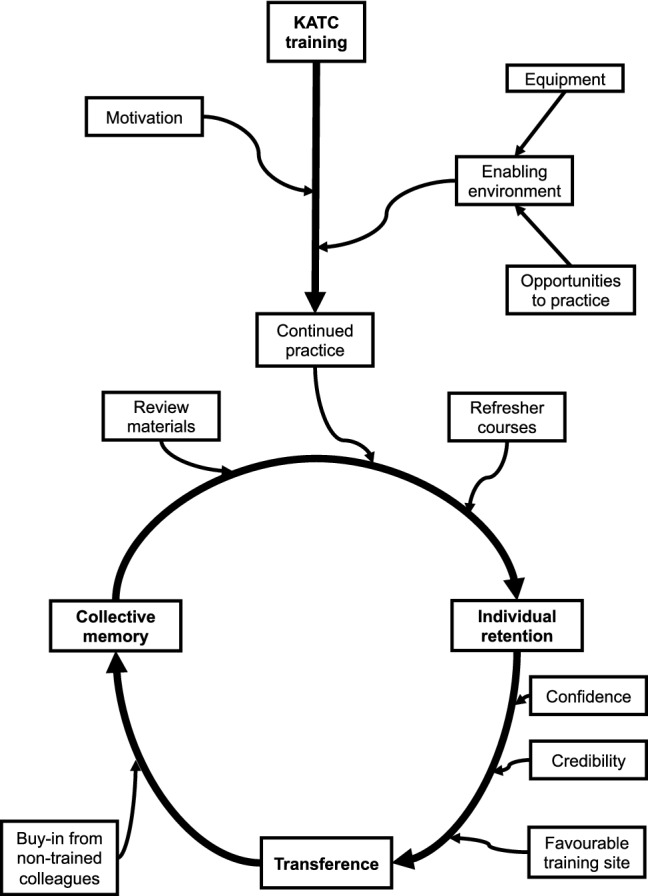
Factors that facilitate retention of trauma training. We constructed a conceptual model of post-course information retention and usage based on interview codes and our own observations. After the trauma course, self-motivation and an enabling environment help providers continue to practice their training. Enabling environments are places where the relevant equipment are available and providers have opportunities to directly participate in trauma management. Once providers feel competent in their own training, they might share their skills with others. Trainers must feel confident about their own abilities, credible from the perspective of other providers and the facility, and have favorable training sites where they have appropriate resources and opportunities to transfer their training. After transference, if other providers continue to use the training, the culture of practice of the facility will change and the training is transferred to the collective memory of all providers. At this stage, the training is no longer dependent on any individual provider and becomes more resilient to decay. Finally, regular practice with review materials and refresher courses help maintain the individual and collective fund of knowledge

We did not observe knowledge transfer between KTPs and non-trained providers. In all facilities, resource-independent practices, such as the “ABCDE” approach and systematic physical examinations, were inconsistently applied. Interview respondents identified two major barriers to wider adoption of best practices: inadequate opportunities to share knowledge with non-trained providers, and reluctance of non-trained providers to adopt unfamiliar practices (Fig. 2).

## Discussion

LMIC trauma programs improve provider confidence and performance on standardized tests [9–11, 17–21]. However, examinations and self-reports are unreliable indicators of competency in motor skills [22, 23] or change in practice. Our study did not find evidence that a trauma training intervention changed trauma practices in home facilities [9].

### Knowledge retention

Training participants, including those in our study, commonly face challenges with knowledge retention [11]. Cognitive aspects of training are more resistant to decay than motor skills, but both decline predictably over time [9–28]. For example, a three-year follow-up survey of 1030 advanced trauma course participants and participant-trainers in India found that less than half retained the training; >   5% wanted refresher courses or to repeat the original course entirely [11]. Trauma courses must include long-term strategies addressing retention. Simulation training, regular facility-sponsored reviews, and team-based training programs are potentially successful follow-up training strategies [29–31].

### Skill transference to non-trained providers

Our study emphasizes the importance of participant selection. We found that large hospitals selected senior surgeons and departmental leaders as participants, but none were responsible for initial trauma management. Consequentially, adoption stagnated: KTPs were not involved in resuscitations and did not transfer knowledge to frontline providers. KTPs became “islands of knowledge,” while frontline providers lacked opportunities to learn guideline-concordant care. Trained frontline providers can change the practices of non-trained peers [10, 11]. Training programs should prioritize frontline providers who are consistently available for trauma management.

### Delays and staff shortages

Faster resuscitation interventions are associated with decreased in-hospital mortality, and completeness and timeliness of evaluations or interventions are important quality indicators for resuscitation [32–34]. Airway, breathing, and circulation management are often described as sequential checkpoints in principle. In practice, well-staffed teams are expected to perform multiple life-saving tasks in parallel [35, 36]. However, in LMIC settings, only one to two providers may be available for resuscitation. Previous work has shown LMIC providers’ adaptability and non-technical skills in response to resource variation [37]. Trauma courses in LMIC must address skills and strategies to overcome common constraints such as limited staffing and equipment.

Our study identified other systemic contributors to trauma care quality. Resource constraints were cited as among the most significant barriers to care, but it is unknown the extent to which improvisation and searching for equipment contribute to delays. While provider ingenuity is integral to trauma response, significant, lasting changes will require institutionally driven solutions to address material and equipment shortages. Second, Uganda lacks a prehospital emergency system, so frontline trauma providers are rarely alerted prior to a patient’s arrival. Implementation of a pre-arrival notification and triage system may improve provider availability and task completion [36, 38]. Finally, more work is needed to characterize and mitigate the impact of patient-to-provider ratios on emergency care.

Our conclusions are limited by small sample sizes. However, we reached conventional sampling thresholds for qualitative research and established thematic saturation. We observed a single work week at each facility, while resuscitation patterns may vary over time. Providers were also aware of our presence during resuscitations. However, it is more likely that reactivity bias would increase adherence to best practices than decrease it. We did not interview non-trained providers who participated in resuscitations. Finally, we did not directly count material and equipment quantities estimated by survey respondents; however, there is broad literature demonstrating poor and variable availability of essential medicines and equipment in Uganda [39, 40].

Our study has several strengths. Trauma training is most appropriately assessed through real-time observations of simulated or actual clinical scenarios. Our mixed-methods strategy enabled us to triangulate evidence on adoption of trauma practices and resources from three distinct sources: direct observation of real practice, in-depth interviews with training participants, and surveys describing resources available for trauma management. Our approach permitted in-depth characterization of perceptions, resources, and actual trauma practices across ten facilities.

## Conclusion

Context-appropriate trauma training programs are popular in LMICs, but incomplete adoption of practices taught in these programs limits their impact (Table 5). We found that barriers to knowledge retention among frontline providers, limited opportunities for skill transfer between trained and non-trained providers, and poor access to necessary materials and equipment limited adoption of best practices for trauma care at facilities that had participated in a trauma training program. We recommend that trauma courses in LMICs prioritize training frontline workers and train more providers from each facility. Simulation training, team training, and refresher courses may be useful adjuncts. Given pervasive staff limitations, trauma training should be offered to frontline cadres, which may include non-physician cadres, and curricula should be adapted to suit these cadres. This will require greater investment in training programs.

**Table 5:** Key messages

Main finding	Potential solutions
Attrition of course training over time due to normal forgetting and limited opportunities to practice	Regularly scheduled re-trainings Increase proportion of trained providers at each facility to build collective knowledge and a culture of practice
Training is not transferred to non-trained providers, and trauma practices are not adopted by the facility as a whole	Train providers who directly work with frontline providers Overlap schedules of trained and non-trained providers
Inconsistent delivery of care due to staffing and resource constraints	Encourage team-based resuscitation whenever possible Help local stakeholders address logistical and infrastructural gaps Advocate at Ministry level to ensure reliability of infrastructure and supplies

## Supplementary Information

Below is the link to the electronic supplementary material.

Supplementary file1 (PDF 579 KB)

Supplementary file2 (DOCX 26 KB)

Supplementary file3 (DOCX 16 KB)

Supplementary file4 (PDF 535 KB)

## Data Availability

The datasets generated during and/or analyzed during the current study are available from the corresponding author upon request.
