# Supplementary material for: Clinical Practices Following Train-The-Trainer Trauma Course Completion in Uganda: A Parallel-Convergent Mixed-Methods Study
Source: World J Surg. 2023 Mar 5;47(6):1399–408. doi: 10.1007/s00268-023-06935-4 (PMC10156777; doi:10.1007/s00268-023-06935-4)
Supplement: Supplementary file 1 — Supplementary file1 (PDF 579 KB) [file 268_2023_6935_MOESM1_ESM.pdf]

Corresponding author: Zeyu Tang, zeyu.tang@yale.edu

Supplementary Table 1. Resuscitation Observation Tool Checklist.

Behavior	Primary Options	Secondary Options
Airway Patency	a. Yes – Airway patent<br>b. Yes – Airway not patent<br>c. Inspection not performed	IF Airway Patency: Yes – Airway not patent, then:<br><br>Definitive Airway:<br>a. Chin-lift/Jaw thrust maneuver<br>b. Endotracheal tube<br>c. Tracheostomy<br>d. Oropharyngeal airway<br>e. Not performed
Clearing airway of foreign bodies or suctioning	a. Performed<br>b. Not Performed	
Ventilation with bag-mask device?	a. Performed<br>b. Not performed	
Supplemental high-concentration oxygen administered?	a. Performed<br>b. Not performed	
Pulse Oximeter attached?	a. Performed<br>b. Not performed	
Check for JVD?	a. Performed<br>b. Not performed	
Check for tracheal deviation?	c. Performed<br>d. Not performed	
Check for breath sounds?	e. Performed<br>f. Not performed	
Chest X-ray	g. Performed<br>h. Not performed	
Hemo/pneumothorax Identified?	i. Yes<br>j. No	IF Hemo/pneumothorax Identified: Yes, then:<br><br>Chest tube placement:<br>a. Performed<br>b. Not performed
Assessment of Pulse	k. Performed<br>l. Not performed	
Two Large-caliber IV Catheters	m. Performed<br>n. Not performed<br>o. One large-caliber IV	
Pelvic binder for hip fracture	a. Performed<br>b. Not performed	
Blood draw for type and screen	c. Performed<br>d. Not performed	
Tourniquets for uncontrolled external hemorrhage	e. Performed<br>f. Not performed	
Pregnancy test for female of childbearing age	g. Performed<br>h. Not performed	
Glasgow Coma Scale evaluation out loud	i. Performed<br>j. Not performed	
Pupillary size and reaction	k. Performed<br>l. Not performed	
Protection of spinal cord with immobilization devices	m. Performed<br>n. Not performed	
Patient deemed unconscious?	o. Yes<br>p. No	IF Patient deemed unconscious: Yes, then:<br><br>Recovery position for unconscious patient:<br>a. Performed<br>b. Not performed
Remove clothing	q. Performed<br>r. Not performed	
Covering with blankets or other warming devices	s. Performed<br>t. Not performed	
Secondary Survey:<br><br>Complete History and Physical	u. Performed<br>v. Not performed	

Corresponding author: Zeyu Tang, zeyu.tang@yale.edu

Secondary Survey:<br><br>Complete Neurological Exam Reperformed	w. Performed<br>x. Not performed	
AMPLE History	y. Allergies<br>z. Medications<br>aa. Past Illnesses<br>bb. Pregnancy<br>cc. Last Meal<br>dd. Events/environment related to injury	
Patient assessed for pelvic fracture?	ee. Exam<br>ff. X-ray<br>gg. CT<br>hh. Not performed	
Patient assessed for pelvic fracture?	ii. Exam<br>jj. Ultrasound<br>kk. CT<br>ll. Not performed	
Neurovascular status of all 4 limbs assessed?	a. Performed<br>b. Not performed	
Patient Characteristics		
Type of trauma	a. Road traffic injury<br>b. Interpersonal violence<br>c. Burn<br>d. Fall<br>e. Other (describe in notes)	
Where was the person injured?	f. Head<br>g. Neck<br>h. Chest<br>i. Abdomen<br>j. Back<br>k. Arms<br>l. Legs	
Gender	m. Male<br>n. Female	
Approximate Age	o. 0 – 14<br>p. 15 – 24<br>q. 24 – 44<br>r. 45 – 65<br>s. 65+	
Sent for surgery?	t. Yes<br>u. No	
Other		
Standard precautions	v. Eye protection<br>w. Face masks<br>x. Water impervious gowns<br>y. Gloves	
Waste management	z. Use of sharps box<br>aa. Use of bins for infectious and non-infectious waste<br>bb. Waste left on floor, trauma tables, or other areas outside the disposal bins	
Foley catheter placement?	cc. Performed<br>dd. Not performed	

Legend: Secondary options are available if specific primary options are checked for a given resuscitation.
