# Supplementary material for: Clinical Practices Following Train-The-Trainer Trauma Course Completion in Uganda: A Parallel-Convergent Mixed-Methods Study
Source: World J Surg. 2023 Mar 5;47(6):1399–408. doi: 10.1007/s00268-023-06935-4 (PMC10156777; doi:10.1007/s00268-023-06935-4)
Supplement: Supplementary file 2 — Supplementary file2 (DOCX 26 KB) [file 268_2023_6935_MOESM2_ESM.docx]

**S2. Kampala Advanced Trauma Care Course Follow Up Survey Interview Questions Guide**

**Question labeled in RED were added in later versions**

**PURPOSE STATEMENT**

The purpose of this study is to explore the perceptions and processes related to trauma care in LMIC among health workers who took part in a trauma training intervention and their immediate colleagues.

Consent:

Thank you for talking with me today. If it’s ok with you, I would like to learn more about your experiences as a health worker performing trauma care in [participant’s healthcare facility]. I’m going to ask you about your feelings about the trauma care equipment and resources, your experiences of providing trauma care, and your experiences with the trauma care learning process. Is that ok with you? [pause]

I would like to record our conversation so that I can remember it more easily later. Only the research team will have access to the interview recordings. If we share anything you said, We will remove your name and other identifying information to protect your privacy. If there is any question you don’t want to answer, you don’t have to answer it. Also, your answers will not affect your relationship with the hospital. Is it ok if I turn on the recorder? [pause]

**Introducing Question:** Please tell me about the traumas you see at your hospital.

- Tell me about a recent trauma case that sticks out in your mind.
- What types of traumas would you say you see the most?
- What do you remember about the traumas that you see?

**Introducing Question: Vignette (hypothetical scenario)**

**There is an early 20s male who has been brought in for a severe boda boda injury in the middle of the day. He is bleeding from the head. He has probably been in the ward for over 30 minutes without any assistance.**

- **What are you thinking about first?**
- **What do you think other health workers are thinking at this time?**
- **How do workers usually react when a trauma comes in?**
- **Why might there be slower responses to trauma cases?**
- **Who do you think should be in charge once a trauma patient comes in?**

**Introducing Question:** How prepared are you for the traumas that you see?

- What are the biggest barriers to trauma care here?
- What kinds of traumas do you think you are the most prepared for? Why?
- What kinds of traumas do you think you are the least prepared for? Why?
- What skills do you think healthcare workers should know to successfully manage traumas?
- **What do you do if there are multiple trauma patients who come in at the same time?**

**Introducing Question:** Please tell me more about the trauma procedure at your hospital.

- What are the strengths and weaknesses of the trauma care in your center?
- What are the best parts of your trauma care?
- How could trauma care in your hospital be improved?
- Who works with you on the traumas? What are their roles? Is there a leader? Do people work together?
- Do you always know your role when you go into a trauma?
- How is the communication between healthcare workers on traumas?
- Are there other support staff available, like social workers?
- How confident are you with your other colleagues that your work with in trauma care?
- How confident do you think your colleagues are in trauma care?
- **In your view, in what situations does teamwork happen?**
- **How do you decide to perform the full primary survey?**

**Introducing Question:** In general, is the equipment you need always available when you are treating traumas?

- How available are the equipment that you need?
- Do you spend any time looking for materials to not find it?
- What happens when you can’t find the equipment that you need? What do you do instead? Can you give an example?
- Have you ever improvised equipment? Why?
- How do you think that has affected your trauma care?
- Are there any equipment that you would like to be available?

## How do you feel about the healthcare system here?

- **What do you or your colleagues do to address issues?**

**Introducing Question:** If you have limited supply, how do you decide whether to use certain equipment or medication on your patients?

- Does your hospital treat patients differently if they are coming in with the same injuries? For example, a child or pregnant woman coming into the hospital versus someone who isn’t?
- Are there resources that you would be able to give to some patients that you wouldn’t give to others? For example, medications or one-time use equipment. For example, depending on time of the month?
  - One patient with tension pneumothorax
  - One patient with simple pneumothorax
  - Both needs chest tubes but we only have one set

## What does it mean to you if you don’t have what you need?

- - - **How does it affect your motivation?**

**Introducing Question:** Are you ever worried about your safety working with trauma patients?

- What traumas make you worried about your safety?
- How do you usually ensure your safety? Example?
- Have you ever seen your colleagues compromise their safety in trauma care? Have you ever seen your colleagues compromise trauma care for safety?
- Are you worried about contracting any diseases from patients or being in the hospital?
- When and what are the personal protective equipment used?
- Gloves, masks, gowns, goggles
- What happens to patients and providers who have needle-stick exposures? How often does it happen?
- Is antiretroviral post-exposure prophylaxis available?

# Trauma Education

**Introducing Question:** How do you learn or improve your trauma care skills currently? (if they do not have an answer, point out the different ways people generally learn about trauma care)

## Do you think nurses should be able to perform the same procedures that doctors can do, if they have the same skills?

- What kind of training was the most helpful?
- Who mentors you in trauma care where you practice from?
- What kind of training do you wish you had more of?
- How did you receive feedback during your training?

## Did you ever perform trauma care before the trauma course? What was that like?

- Are the training materials you practiced with during the course the same as the ones in the hospital?
- what difference do notice in yourself after the trauma course?
- Do you notice any differences in your colleagues who have taken the trauma course?
- What proportion of your colleagues would you say have received trauma training?
- How has the portable reference book been used?
- How do you think the training has changed patient outcomes?
- How much of the trauma course training would you say you consistently use?
- How does the hospital respond to medically preventable deaths?
- **Have you attended any other trainings aside from the KATC? What has been the impact of these trainings on patient care?**

# Conclusion

**Introducing Question:** That’s all the questions I have. Is there anything we haven’t talked about that you think is important?

- Is there anything you want to ask me?
