# Supplementary material for: Clinical Practices Following Train-The-Trainer Trauma Course Completion in Uganda: A Parallel-Convergent Mixed-Methods Study
Source: World J Surg. 2023 Mar 5;47(6):1399–408. doi: 10.1007/s00268-023-06935-4 (PMC10156777; doi:10.1007/s00268-023-06935-4)
Supplement: Supplementary file 3 — Supplementary file3 (DOCX 16 KB) [file 268_2023_6935_MOESM3_ESM.docx]

Clinical Practices Following Train-The-Trainer Trauma Course Completion in Uganda: A Parallel-Convergent Mixed-Methods Study World Journal of Surgery

Corresponding author: Zeyu Tang, [zeyu.tang@yale.edu](mailto:zeyu.tang@yale.edu)

Supplementary Table 3. Sample Quotations of Interviews for Thematic Analysis

| **Sample Excerpts** | |
| --- | --- |
| A. Overall Impression | **Nurse, tertiary public facility:** We used to handle patients as they would come. But at the end of it all sometimes, you would find you are missing out the basics but when the trauma training team came they highlighted us on the key things you have to look for while assessing the patients, especially when you have like massive trauma casualties. Being on the roadside we normally get sometimes massive trauma casualties and they pour like thirty at a go, so sometimes you would start on who is closer to you, but when the trauma training course came in, they taught us how to identify those potentially dangerous patients whom we can lose in the quickest short time and it has  improved our basics especially in triaging who’s most sick, who needs urgent attention. |
| B. Forgetting | **Medical clinical officer, district:** Yeah, I think the issue is they forget. You know when you don’t do it regularly, so sometimes when the patient comes, you tend to go to the old thing because maybe you don’t have someone close to you who can help, who’s also trained who has the same skills with you. So that you flow. So you end up doing something different though you can command here and think we have to do, so it’s something that we need to, to make it wide so that people get to know that it is fast and it can save life  better than the other old one. |
| C. “Islands of Knowledge” | **Specialist surgeon, tertiary public facility:** Right now there are islands of knowledge where a few people have the skills for trauma care but not everyone does. |
|  | **Senior house officer, tertiary public facility:** So far the training that is being done is haphazard… it’s a drop in an ocean I should say. It is  something but really, if, if for instance from the whole Casualty Department, you train one doctor and one nurse… Those two don’t form a trauma team. So, via knowledge, yes they have acquired the skill but the impact of their knowledge might not really give measurable outcomes, because they don’t have, they don’t have a team with which they can work and they cannot be  there all the time. |
| D. Gaps in Coverage | **Medical officer, tertiary public facility:** [Intern doctors] temporarily stay in the department so in a few months, we have a new group of intern doctors coming in, so when you pass on the skill to those very doctors, they, they’ll not be there in a few, after four or three months, they’re, they’re gone. So that, that becomes a challenge. The same to the nursing officers or the nurses, if you pass on the skill to one or two, after a few months, they are in another  ward or in another department… So we don’t have permanent staff. |
| E. Resource Inadequacies | **Senior house officer, tertiary public facility:** The institution has its own challenges and so you have trained the individual but what will the single individual do in a big facility like mine? Where will you start from? The challenges of handling a trauma are so imbedded in the weaknesses or the challenges of the institution, that even the knowledge you have acquired yes, it can help at the moment when you are the doctor attending to this patient that has come in, but as in improving trauma care generally, it almost doesn’t  help. |
