# Supplementary material for: Clinical Practices Following Train-The-Trainer Trauma Course Completion in Uganda: A Parallel-Convergent Mixed-Methods Study
Source: World J Surg. 2023 Mar 5;47(6):1399–408. doi: 10.1007/s00268-023-06935-4 (PMC10156777; doi:10.1007/s00268-023-06935-4)
Supplement: Supplementary file 4 — Supplementary file4 (PDF 535 KB) [file 268_2023_6935_MOESM4_ESM.pdf]

Corresponding author: Zeyu Tang, zeyu.tang@yale.edu

Supplementary Table 4. Resource Access Survey Results

Criterion	Private Not-for-profit (N = 4)	Public Tertiary (N = 15)	District-level (N = 10)
**Overall Rating (Max = 222)**	**180**	**98**	**108**
**Airway**			
Basic trauma pack	2 (0 - 3)	0.5 (0 - 2)	0.5 (0 - 3)
Capnography	0 (0 - 3)	0 (0 - 1)	0 (0 - 0)
Cricothyroidotomy set	0 (0 - 3)	0 (0 - 1)	0 (0 - 2)
Endotracheal tubes (adult)	3 (1 - 3)	2 (0 - 3)	2 (0 - 3)
Endotracheal tubes (pediatric)	3 (0 - 3)	1.5 (0 - 3)	1 (0 - 3)
Laryngoscope handle	3 (0 - 3)	1 (0 - 3)	0.5 (0 - 3)
Laryngoscope Macintosh blades (adult) with bulbs and batteries	3 (0 - 3)	1 (0 - 3)	0 (0 - 3)
Magill forceps (adult)	2 (0 - 3)	0 (0 - 2)	1 (0 - 3)
Magill forceps (pediatric)	0.5 (0 - 3)	0 (0 - 1)	0 (0 - 1)
Esophageal detector device	0 (0 - 1)	0 (0 - 1)	0 (0 - 1)
Oral or nasal airway	3 (2 - 3)	1 (0 - 2)	2 (0 - 3)
Oropharyngeal airway (adult size)	3 (2 - 3)	1 (0 - 3)	2 (0 - 3)
Oropharyngeal airway (pediatric size)	2.5 (1 - 3)	1 (0 - 3)	1 (0 - 2)
Resuscitator bag valve and mask (adult)	3 (3 - 3)	2 (1 - 3)	3 (0 - 3)
Resuscitator bag valve and mask (pediatric)	3 (2 - 3)	1 (0 - 3)	2 (0 - 3)
Suction device: at least manual (bulb) or foot pump	3 (0 - 3)	1 (0 - 3)	2 (0 - 3)
Suction device: powered: electric/pneumatic	3 (3 - 3)	2 (1 - 3)	0 (0 - 3)
Suction tubing	3 (3 - 3)	2 (1 - 3)	1 (0 - 3)
Yankauer or other stiff suction tip	3 (1 - 3)	1 (0 - 3)	0 (0 - 2)
**Breathing**			
Arterial blood gas measurements	2.5 (0 - 3)	0 (0 - 2)	0 (0 - 3)
Chest tubes	2 (1 - 3)	1 (0 - 3)	0.5 (0 - 2)
Mechanical ventilator	2 (0 - 3)	0 (0 - 1)	0 (0 - 2)
Nasal prongs, face mask, associated tubing	3 (3 - 3)	2 (1 - 3)	2 (0 - 3)
Needle & syringe	3 (3 - 3)	3 (2 - 3)	3 (2 - 3)

Oxygen supply (cylinder, concentrator or other source)	3	(3 - 3)	2	(1 - 3)	2	(0 - 3)
Pulse oximetry	3	(3 - 3)	2	(0 - 3)	1	(0 - 3)
Stethoscope	3	(3 - 3)	2	(1 - 3)	2.5	(1 - 3)
Underwater seal bottle (or equivalent)	2	(1 - 3)	1	(0 - 3)	0.5	(0 - 3)
**Circulation**						
Arterial tourniquet in extreme situations	3	(1 - 3)	1	(0 - 3)	3	(0 - 3)
Blood pressure (BP) cuff	3	(3 - 3)	2	(1 - 3)	3	(1 - 3)
Blood transfusion capabilities	3	(3 - 3)	2	(1 - 3)	2.5	(0 - 3)
Central venous lines	2	(1 - 3)	0	(0 - 1)	0.5	(0 - 3)
Clock or watch with second hand	3	(1 - 3)	1	(0 - 3)	2.5	(1 - 3)
Colloids	3	(3 - 3)	2	(0 - 3)	1	(0 - 3)
Crystalloid	3	(3 - 3)	2	(0 - 3)	2	(0 - 3)
Electronic cardiac monitoring	3	(1 - 3)	0	(0 - 3)	0	(0 - 0)
Fluid warmers	3	(1 - 3)	1	(0 - 3)	0	(0 - 3)
Gauze and bandages	3	(3 - 3)	2	(1 - 3)	2	(1 - 3)
Intraosseous needle or equivalent	2	(1 - 3)	0.5	(0 - 3)	0	(0 - 3)
Intravenous infusion set (lines and cannulas)	3	(3 - 3)	3	(2 - 3)	3	(2 - 3)
Laboratory facilities for arterial blood gases	3	(3 - 3)	0	(0 - 2)	0	(0 - 3)
Laboratory facilities for electrolytes	3	(3 - 3)	2	(0 - 3)	1	(0 - 3)
Laboratory facilities for haemoglobin or haematocrit	3	(3 - 3)	2	(0 - 3)	2	(1 - 3)
Laboratory facilities for lactate	3	(3 - 3)	0	(0 - 1)	0	(0 - 1)
Monitoring of central venous pressure	0	(0 - 0)	0	(0 - 1)	0	(0 - 2)
Nasogastric (NG) tube	3	(3 - 3)	2	(2 - 3)	2	(0 - 3)
Pressors (for neurogenic/spinal shock)	3	(1 - 3)	0	(0 - 2)	0	(0 - 3)
Right-heart catheterization	0	(0 - 0)	0	(0 - 0)	0	(0 - 0)
**Disability**						
Cervical Collar	3	(2 - 3)	1	(0 - 3)	0	(0 - 1)
Spine Back Board	3	(0 - 3)	0	(0 - 2)	0	(0 - 0)
**General**						
Best practice guidelines for emergency care	3	(1 - 3)	1	(0 - 2)	1	(0 - 3)
Forcep, artery	3	(3 - 3)	3	(1 - 3)	2	(1 - 3)

Corresponding author: Zeyu Tang, zeyu.tang@yale.edu

Intravenous cannulas/scalp vein infusion set	3	(1 - 3)	2	(0 - 3)	2.5	(0 - 3)
Inventory list of equipment and supplies	3	(1 - 3)	1	(0 - 3)	3	(1 - 3)
Light source (lamp and flashlight)	3	(1 - 3)	1	(0 - 3)	3	(0 - 3)
Needle holder	3	(3 - 3)	2	(1 - 3)	2	(1 - 3)
Retractor	1.5	(0 - 3)	1	(0 - 2)	2	(0 - 3)
Scalpel with blades	3	(0 - 3)	2	(1 - 3)	2	(0 - 3)
Scissors	3	(3 - 3)	2	(1 - 3)	2.5	(1 - 3)
Sterilizer	3	(3 - 3)	2	(0 - 3)	2.5	(1 - 3)
Thermometer	3	(3 - 3)	1	(0 - 3)	3	(1 - 3)
Urinary catheter	3	(3 - 3)	2	(1 - 3)	3	(1 - 3)
Vaginal Speculum	3	(1 - 3)	1	(0 - 3)	3	(0 - 3)
Weighing scale for children	1.5	(0 - 3)	0	(0 - 2)	0	(0 - 3)
**Safety**						
Eye protection	3	(1 - 3)	0	(0 - 1)	0.5	(0 - 3)
Face masks	3	(3 - 3)	1	(0 - 3)	2.5	(1 - 3)
Gloves (examination)	3	(3 - 3)	2	(1 - 3)	2.5	(2 - 3)
Gloves (sterile)	3	(3 - 3)	2	(1 - 3)	3	(2 - 3)
Needles and sutures	3	(3 - 3)	2	(1 - 3)	2.5	(1 - 3)
Protective gowns/aprons	3	(2 - 3)	1	(0 - 3)	3	(0 - 3)
Sharps disposal container	3	(3 - 3)	3	(1 - 3)	3	(2 - 3)
Soap	3	(3 - 3)	2	(1 - 3)	2	(1 - 3)
Splints for arm, leg	3	(1 - 3)	1	(0 - 3)	1.5	(0 - 3)
Waste disposal container	3	(3 - 3)	3	(1 - 3)	3	(2 - 3)

Legend: Score for each item is listed as an aggregate of providers in each facility type, displayed as median (range).
